# The Importance of Screening for Adverse Childhood Experiences (ACE) in all Medical Encounters

**DOI:** 10.1016/j.focus.2023.100131

**Published:** 2023-06-25

**Authors:** Jeoffry B. Gordon, Vincent J. Felitti

**Affiliations:** 1 California Citizens Review Panel on Critical Incidents, Sacramento, California; 2 Emeritus, University of California School of Medicine, San Diego, California; 3 Emeritus, Department of Preventive Medicine, Kaiser Permanente Medical Group, San Diego, California

The practical, insightful value of adverse childhood experience (ACE) screening of individuals in the clinic to preventive and clinical medicine is profound. There are more than 600,000 substantiated cases of child abuse or neglect (CAN) each year in the U.S., including at least 1,700 child maltreatment homicides annually^1^—about as many deaths as are caused by childhood cancer. In children aged <18 years, the population prevalence of identified CAN is at least 1 in 8.^2^ Among U.S. adults living in the community, the prevalence of an ACE score of 4 or more is 15.6% or 1 in 6 adults.^3^ An ACE score is not a diagnosis but is a proven^4^ screen for assessing CAN categories, which contribute to many physical and mental illnesses.^3^ The ACE score reflects the number of categories (not events) of adverse childhood events experienced in the first 18 years of life. Many but not all persons who experience the sentinel circumstances enumerated by the ACE score will develop damaging outcomes. The effects of CAN trauma can be mitigated by positive childhood experiences, which strengthen integrity and resilience.^5^^,^^6^ Child welfare social services are evolving to emphasize promoting positive experiences in at-risk families with children. The biomedical and economic benefits of ACE screening have been sufficient to result in supportive legislation in 39 states and the District of Columbia as of 2021.^7^

Especially in pediatrics, the routine ACE screen has the red flag significance of identifying concurrent CAN or suicidal thoughts or intent. The potential value of routine ACE screening in adults has been illustrated by the epidemic of children sexually abused as Boy Scouts. Many victims only disclosed their abuse to others after a 29-year delay, with an average age at disclosure of 42 years, with 50% of the victims then aged ≥50 years.^8^ According to a Centers for Disease Control and Prevention analysis,^3^ significant CAN trauma is associated with substantial mental, physical, and social illness and disease. Nonetheless, the vast majority of medical practitioners in all specialties do not fully appreciate the prevalence of child abuse trauma^9^ nor its dose–response association with mental and physical illness, especially among adults.^10^ Merrick et al.^3^ document that a substantial proportion of people with high ACE scores are current smokers and/or heavy drinkers. Inspired by the California Positive and Adverse Experience (PACEs) stakeholders, the California Department of Public Health published the first tobacco cessation treatment manual integrating ACE knowledge into therapy with their publication “Trauma-Informed Approaches to Tobacco Prevention and Cessation.”^11^

In the clinical setting, questions about domestic violence, suicidal intentions, and sexual abuse have all been perceived as upsetting or triggering by physicians even though they are now accepted as necessary for quality care. Such concerns reflect discomfort by clinicians rather than by patients. Felitti and his department initially had to overcome medical staff hesitancy but then had no negative experiences in screening 440,000 adult patients^12^ undergoing a comprehensive medical evaluation. Indeed, it was common to hear spontaneous patient expressions of appreciation for the opportunity to open up for the first time about their ACE, creating new intimacy in the doctor–patient relationship. Any upset patient or negative response may indicate the need for a more relaxed, skilled approach. Indeed, it is more upsetting when a patient's screening reveals trauma exposure, and the clinician does not respond with an appropriate and sensitive exploratory inquiry.^13^

An important aspect of any screening is that it leads to effective treatment. The underlying contributions to disease and psychosocial distress uncovered by ACE screening expose complex biopsychosocial medical problems that may challenge the clinical practitioner unless previous attention is given to planning an efficient appropriate therapeutic response. This must include a trauma-informed care perspective^14^ and a comprehensive medical history assessing both mental and physical illness. Although psychiatry has yet to recognize CAN trauma as a diagnosable condition,^15^ optimal treatment requires specialized behavioral and social therapies. Multiple compilations of evidence-based treatments and resources are available^16^ and routinely taught and widely used. Unfortunately, many physicians are isolated from easy referrals to behavioral health and community treatment resources and have not developed their own experience in this realm. Competent specialized interdisciplinary resources (although often overburdened) are available across the U.S. and have been compiled to assist clinicians in finding treatment for these patients and their families.^17^ Because all clinicians are mandated reporters, the causal diagnosis may have challenging reporting and legal consequences, especially in children. This reinforces the importance of the integration of mental health and social work resources into the clinic.

ACE screening of individual patients should not be avoided owing to the guidance from the American College of Preventive Medicine (ACPM), recently published as “Recommendations for Population-Based Applications of the Adverse Childhood Experiences Study: Position Statement by the American College of Preventive Medicine.”^18^ Although the ACPM acknowledges the profound, well-documented, substantial adverse sequelae of CAN and states that “interventions to mitigate its harmful effects are essential” and recognizes that “evidence is emerging that ACEs are both a cause and a consequence of health disparities,” the “ACPM recommends against individual ACE screening in clinical settings.” This ACPM position statement creates a perceived incentive for many clinicians to avoid addressing child abuse and neglect, its antecedents, and its consequences, including its many lifelong physical, mental, socioeconomic, and racial comorbidities. For the many reasons described earlier, as clinicians, we, among many others, have found the individual ACE screen to be important, practical, functional, and acceptable in the clinic. Furthermore, the reputation of the ACE screen makes it the perfect tool for motivating most medical practitioners—who have yet to recognize the clinical significance of child maltreatment across the life span^19^—to introduce this risk assessment into routine practice with the appreciation that CAN trauma makes a substantial contribution to morbidity, making this knowledge so relevant to effective treatment.

## CRediT authorship contribution statement

**Jeoffry B. Gordon:** Conceptualization. **Vincent J. Felitti:** Conceptualization, Writing – review & editing.

## References

[1] U.S. Department of Health & Human Services (2022).

[2] Wildeman C, Emanuel N, Leventhal JM (2014). The prevalence of confirmed maltreatment among US children, 2004 to 2011. JAMA Pediatr.

[3] Merrick MT, Ford DC, Ports KA (2019). Vital Signs: Estimated proportion of adult health problems attributable to adverse childhood experiences and implications for prevention — 25 states, 2015–2017. MMWR. Morbidity and Mortality Weekly Report.

[4] Mei X, Li J, Li ZS (2022). Psychometric evaluation of an Adverse Childhood Experiences (ACEs) measurement tool: an equitable assessment or reinforcing biases?. Health Justice.

[5] Merrick JS, Narayan AJ. (2020). Assessment and screening of positive childhood experiences along with childhood adversity in research, practice, and policy. J Child Pover.

[6] Bethell C, Jones J, Gombojav N, Linkenbach J, Sege R. (2019). Positive childhood experiences and adult mental and relational health in a statewide sample: associations across adverse childhood experiences levels. JAMA Pediatr.

[7] State ACEs and TI Laws and Resolutions Clickable Map https://www.pacesconnection.com/g/state-aces-action-group/pages/clickable-map-aces-and-ti-laws-and-resolutions. Accessed December 10, 2022.

[8] Hamilton MA, Carter E, Timon CE. Scouting abuse: analysis of victims experiences, Part I. Available at www.CHILDUSA.org. Accessed July 11, 2023.

[9] Stork BR, Akselberg NJ, Qin Y, Miller DC. (2020). Adverse Childhood Experiences (ACEs) and community physicians: what We've learned. Perm J.

[10] Gordon JB. (2021). The importance of child abuse and neglect in adult medicine. Pharmacol Biochem Behav.

[11] *Trauma-Informed Approaches to Tobacco Prevention and Cessation*; 2022. California Department of Public Health, Injury and Violence Prevention Branch, the California Department of Social Services, Office of Child Abuse Prevention. *California Essentials for Childhood Initiative*. CA: California Department of Public Health, California Department of Social Services. www.ca.tccc.org.

[12] Felitti VJ, Anda RF, Chadwick DL, Giardino AP, Alexander R, Thackeray JD, Esernio-Jenssen D (2014). Child Maltreatment.

[13] Austin AE. (2021). Screening for traumatic experiences in health care settings: a personal perspective from a trauma survivor. JAMA Intern Med.

[14] Substance Abuse and Mental Health Services Administration (2014).

[15] (2013). https://www.psychdb.com/teaching/dsm-v-icd-z-codes.

[16] For instance, see Evidence-Based Treatments Addressing Trauma, Trauma Informed Care: Perspectives and Resources, JBS International and Georgetown University National Technical Assistance Center for Children's Mental Health, Georgetown University Center for Child and Human Development, https://gucchd.georgetown.edu/TraumaInformedCare/Module4.html. Accessed July 11, 2023

[17] See https://www.childwelfare.gov/aboutus/find-help/ or findtreatment.gov https://www.samhsa.gov/find-treatment or https://www.all4kids.org/tools-and-resources/. Accessed July 11, 2023.

[18] Sherin KM, Stillerman AJ, Chandrasekar L, Went NS, Niebuhr DW. (2022). Recommendations for population-based applications of the adverse childhood experiences study: position statement by the American College of Preventive Medicine. AJPM Focus.

[19] Bhushan D, Kotz K, McCall J (2020).

